# Age‐specific prevalence and determinants of depression in long‐term breast cancer survivors compared to female population controls

**DOI:** 10.1002/cam4.3476

**Published:** 2020-10-06

**Authors:** Daniela Doege, Melissa S. Y. Thong, Lena Koch‐Gallenkamp, Lina Jansen, Heike Bertram, Andrea Eberle, Bernd Holleczek, Ron Pritzkuleit, Annika Waldmann, Sylke R. Zeissig, Hermann Brenner, Volker Arndt

**Affiliations:** ^1^ Unit of Cancer Survivorship Division of Clinical Epidemiology and Aging Research German Cancer Research Center (DKFZ) Heidelberg Germany; ^2^ Division of Clinical Epidemiology and Aging Research German Cancer Research Center (DKFZ) Heidelberg Germany; ^3^ Cancer Registry of North Rhine‐Westphalia Bochum Germany; ^4^ Bremen Cancer Registry Leibniz Institute for Prevention Research and Epidemiology ‐ BIPS Bremen Germany; ^5^ Saarland Cancer Registry Saarbrücken Germany; ^6^ Cancer Registry of Schleswig‐Holstein Lübeck Germany; ^7^ Hamburg Cancer Registry Hamburg Germany; ^8^ Institute of Social Medicine and Epidemiology University Lübeck Lübeck Germany; ^9^ Cancer Registry of Rhineland‐Palatinate Mainz Germany; ^10^ Division of Preventive Oncology German Cancer Research Center (DKFZ) Heidelberg Germany; ^11^ German Cancer Consortium (DKTK) German Cancer Research Center (DKFZ) Heidelberg Germany

**Keywords:** age factors, breast cancer, depression, long‐term cancer survivors, mental health, population health

## Abstract

**Background:**

Depression is more prevalent in breast cancer (BC) survivors than in the general population. However, little is known about depression in long‐term survivors. Study objectives were: (1) to compare the age‐specific prevalence of depressive symptoms (a) in BC survivors vs female population controls, (b) in disease‐free BC survivors vs BC survivors with self‐reported recurrence vs controls, and (2) to explore determinants of depression in BC survivors.

**Methods:**

About 3010 BC survivors (stage I‐III, 5‐16 years post‐diagnosis), and 1005 population controls were recruited in German multi‐regional population‐based studies. Depression was assessed by the Geriatric Depression Scale‐15. Prevalence of mild/severe and severe depression only were estimated via logistic regression, controlling for age and education. Multinomial logistic regression was used to explore determinants of mild and severe depression.

**Results:**

Compared with population controls, BC survivors were more likely to report mild/severe depression (30.4% vs 23.8%, *p* = .0003), adjusted for age and education. At all age groups <80 years, prevalence of both mild/severe and severe depression only was significantly higher in BC survivors, while BC survivors ≥80 years reported severe depression less frequently than controls. BC survivors with recurrence reported significantly higher prevalence of mild/severe depression than disease‐free survivors and controls, but prevalence in disease‐free survivors and controls was comparable. Age, income, living independently, recurrence, and BMI were significant determinants of mild depression in BC survivors. Age, education, employment, income, recurrence, and BMI were significant determinants of severe depression.

**Conclusions:**

Long‐term BC survivors <80 years report significantly higher prevalence of depressive symptoms than controls, which might be explained by recurrence and individual factors. The findings suggest that depression in BC survivors is common, and even more after BC recurrence. Clinicians should routinize screening and normalize referral to psychological care.

## Graphical abstract

Long‐term breast cancer survivors (5‐16 years post‐diagnosis) with recurrence reported significantly higher prevalence of mild/severe depression than disease‐free survivors and controls, but prevalence in disease‐free survivors and controls was comparable. Cancer recurrence, age, education, employment, income, living independently, and BMI were significant determinants of depression in breast cancer survivors.

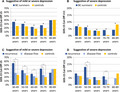

## 1 BACKGROUND

Breast cancer (BC) is the most commonly diagnosed cancer in women, with more than 2 million new cases worldwide in 2018.^1^ Better prognosis and demographic aging lead to a steadily increasing number of long‐term cancer survivors (≥5 years post‐diagnosis).^2^ However, persisting detriments in health‐related quality of life can still be found in long‐term survivors, resulting in lower functioning and higher symptom burden compared to general population.^3^, ^4^, ^5^ These lingering problems are associated with psychological distress and may, therefore, represent risk factors for the development of affective disorders.^6^

A systematic review analyzed the prevalence of depression in short‐term BC survivors (on average 3.9 years post‐diagnosis), finding prevalence rates of 9% to 66% for any symptoms of depression and of 3% to 42% for severe symptoms of depression.^7^ The wide range might be due to differences in time since diagnosis, depression scale, and severity level.^7^

Only a small number of cross‐sectional studies have looked at depressive symptoms in long‐term BC survivors.^8^ A US study found that 13% of the included disease‐free BC survivors (5‐10 years post‐diagnosis) met clinical definitions of being depressed,^9^ but no control group was involved. In a German study, 17% of survivors of different cancers 5‐10 years post‐diagnosis reported moderate to severe depression, with BC survivors reporting the highest mean scores.^10^ In comparison to the general population, cancer survivors <70 years were more depressed than same‐aged controls in this study, while there was no difference at older age.^10^ Comparison to age‐matched controls is crucial as mild depression is generally more frequent at higher age,^11^ but is only considered in a few studies.

Further, only few studies in long‐term BC survivors have looked at determinants of depression. Being overweight or obese are reciprocally associated with depression.^12^ Specifically in women with BC shortly after treatment, personal factors (eg, partnership, having children) and social factors (socioeconomic status, social contact),^13^ treatment‐related variables,^13^ and poor body image were associated with depression, with the latter factor even more so when the partner shows little empathy.^14^ It is of interest to identify which factors are associated with depression in long‐term BC survivors and whether these factors differ in same‐aged controls. Such information will assist to determine whether specific prevention strategies are needed for BC survivors.

Pertinent studies also did not account for different disease trajectories such as remission and recurrence states.^15^ Recurrence in BC is associated with distress, hopelessness, and impairments in physical, functional, and emotional well‐being.^16^, ^17^ These factors can also be related to depression.

The objectives of this population‐based study are to compare the prevalence of depression in (1) BC long‐term survivors (stage I to III at primary diagnosis) vs female general population controls, (2) BC long‐term survivors who remained disease‐free after treatment vs those with self‐report of recurrence, and (3) to assess potential factors associated with depression in BC survivors and controls.

## 2 METHODS

### 2.1 Study population

Participants were recruited in two multi‐regional population‐based studies in Germany, CAESAR+ (“Cancer Survivorship—A multi‐regional population‐based study”) and LINDE (“Lebensqualität in Deutschland”—Quality of life in Germany). All procedures were performed after approval by the responsible institutional ethics committees. Written informed consent was obtained from each participant.

#### 2.1.1 CAESAR+ (BC survivors)

The CAESAR+ study included long‐term breast, colorectal, and prostate cancer survivors diagnosed between 1994 and 2004, and reported to one of six participating German cancer registries (Bremen, Hamburg, North Rhine‐Westphalia, Rhineland‐Palatinate, Saarland, and Schleswig‐Holstein).^18^ Inclusion criteria were age at diagnosis 20‐75 years and a histological confirmation of the cancer. Participants answered postal questionnaires between March 2008 and May 2011. Non‐respondents received up to two reminder letters and a telephone contact. Out of the 6553 BC survivors contacted, 3045 completed the full‐length questionnaire (response rate: 46.5%) and were eligible for the present analysis. Respondents with stage IV (M1) at primary diagnosis (n = 35) were excluded in this analysis, leading to a final sample of 3010 BC survivors (Figure S1).

#### 2.1.2 LINDE (population controls)

The LINDE study aimed to provide reference values on a range of patient‐reported outcomes from a representative sample of the German population. A total of 10,580 men and women, aged 18 and above, stratified by age and sex, were randomly selected from the general German population via regional municipal offices. Data were collected between 2013 and 2014 by postal questionnaire. Non‐respondents received two follow‐up reminder letters and a telephone contact (or one mailed reminder and a home visit, if necessary). In total, 2849 individuals participated (response rate: 29%). As a comparison group for BC survivors, only females who completed the full‐length questionnaire (n = 1306) were included. Women with self‐reported history of cancer (n = 165) and who were younger (<30 years, n = 124) or older than the BC sample (>89 years, n = 12) were excluded for this analysis. The final sample comprised 1005 female LINDE participants.

### 2.2 Measurements

#### 2.2.1 Depression

Depression was assessed by the German short form *Geriatric Depression Scale* (GDS‐15)^19^, ^20^ in both samples. The GDS‐15 is a screening instrument with 15 dichotomous items and a resulting sum score of 0‐15. Of the different proposed cut‐off scores,^21^ we used the following^22^: Sum score <5 no depression, ≥5 suggestive of a mild depression (“mild depression”), ≥11 suggestive of a severe depression (“severe depression”). The GDS‐15 was chosen with respect to the mean age of the elderly sample. In contrast to other depression screening instruments, the yes‐no‐items are thought to be easy to understand and the scale can be answered in a short time.^19^

#### 2.2.2 Sociodemographic and clinical data

In both studies, the questionnaires included sociodemographic, personal, and clinical information such as marital status, education, income, comorbidities, weight, and body size. In BC survivors, information on treatment and on recurrence, metastasis or new cancer (“disease recurrence”) since initial diagnosis were also assessed via self‐report. The particular cancer registries provided additional clinical information on cancer survivors such as year of diagnosis and cancer stage.

### 2.3 Statistical analysis

Differences between the characteristics of BC survivors and controls were evaluated with Cochran‐Mantel‐Haenszel tests (CMH). Dummy variables were created for each characteristic to allow for a comparison by variable level and to allow for age standardization. The age distribution of population controls reflected a stratified sampling scheme but was still significantly different from that of BC survivors (mean age 58.7 vs 65.3 years). Consequently, for the comparison of further characteristics, we used direct standardization by age (categorized as 30‐49, 50‐59, 60‐69, 70‐79, and 80‐89 years) to adjust the age distribution of population controls to that of BC survivors. The comparison of BC survivors with and without recurrence was also done using dummy variables and CMH tests, but without age standardization.

Prevalence of mild/severe and severe depression only (according to GDS cut‐off‐scores) was estimated via logistic regression, controlling for age and education by including these factors in all models (age: categorized as 30‐54, 55‐59, 60‐64, 65‐69, 70‐74, 75‐79, 80‐84, and 85‐89 years, education: categorized as ≤9 years, 10‐11 years, ≥12 years). Contrasts were estimated to compare subgroups. Mild depression was not analyzed as intermediate distinct category in subgroup comparison, as otherwise a higher prevalence of severe depression in one group could have led to a lower prevalence of mild depression in this group. Employment status and comorbidity also differed between BC survivors and controls, but were not included for adjustment, as they reflect the situation at the time of the survey and differences could also be cancer‐related.

For age‐stratified comparison of depression prevalence, age at the survey was categorized as follows: 30‐49, 50‐59, 60‐69, 70‐79, and 80‐89 years. Further stratification was done by self‐reported recurrence status of BC survivors at survey (disease recurrence vs disease‐free).

Multinomial logistic regression was applied to explore determinants of mild and severe depression in BC survivors and controls. Both outcomes were modeled simultaneously. The regression model for controls comprised the same factors as the model for BC survivors, except for the cancer‐related variables.

We employed multiple imputation, based on the Markov Chain Monte Carlo method with 25 repetitions, to reduce possible bias due to missing values (in general less than 10%). All analyses were conducted with SAS (version 9.4 for Windows; SAS Institute Inc). A *p*‐value <.05 (two‐sided) was considered statistically significant.

## 3 RESULTS

### 3.1 Non‑respondent analysis

As reported previously, respondents and non‐respondents of the CAESAR+ study were comparable on most variables, except that respondents were slightly younger at diagnosis, had a shorter time since diagnosis, and were less likely to have distant metastasis/ stage IV disease.^5^

### 3.2 Study population characteristics

Even after age standardization, BC survivors reported lower education (≤9 years of education: 54.3% vs 43.5%; ≥12 years: 17.6% vs 25.2%), were less likely to work full‐time (7.5% vs 13.6%), and were more often retired at the time of the survey (49.9% vs 43.8%) in comparison to controls. BC survivors reported higher proportions of heart failure (9.3% vs 6.4%) and ever having had depression (22.9% vs 18.9%). There were no differences in having a partner or children (Table 1).

**TABLE 1:** Description of breast cancer (BC) survivors (stage I to III at diagnosis) and population controls

	BC survivors		Population controls			Diff. unadj.^b^		Diff. adj.^b^	
	n	%	n	%	% adj.^a^	%	*P* (CMH)	%	*P* (CMH)
Total	3010	100.0	1005	100.0					
Mean age (SD)	65.3	(9.6)	58.7	(14.3)		4.7	**<0.0001**		
Age at survey									
30‐49	216	7.2	309	30.7	7.2	−23.5	**<0.0001**	—	—
50‐59	597	19.8	222	22.1	19.8	−2.3	0.12	—	—
60‐69	1083	36.0	209	20.8	36.0	15.2	**<0.0001**	—	—
70‐79	962	32.0	160	15.9	32.0	16.1	**<0.0001**	—	—
80‐89	152	5.0	105	10.4	5.0	−5.4	**<0.0001**	—	—
Education									
≤9 y	1633	54.3	347	34.5	43.5	19.8	**<0.0001**	10.7	**<0.0001**
10 y	848	28.2	339	33.7	31.3	−5.5	**0.001**	−3.1	0.12
≥12 y	529	17.6	319	31.8	25.2	−14.2	**<0.0001**	−7.6	**<0.0001**
Employment									
Full‐time	225	7.5	207	20.6	13.6	−13.1	**<0.0001**	−6.1	**<0.0001**
Part‐time	434	14.4	259	25.8	16.9	−11.4	**<0.0001**	−2.5	0.05
Unemployed	47	1.6	28	2.8	2.4	−1.2	**0.0253**	−0.8	0.16
Housewife	694	23.1	181	18.0	20.1	5.1	**0.0010**	3.0	0.10
(Early) Retirement	1503	49.9	288	28.6	43.8	21.3	**<0.0001**	6.1	**0.0015**
Other	106	3.5	42	4.2	3.2	−0.7	0.36	0.3	0.63
Having a partner	2141	71.1	726	72.2	71.0	−1.1	0.52	0.1	0.88
Having children	2556	84.9	833	82.9	85.5	2.0	0.13	−0.6	0.67
Comorbidities (self‐report)									
Stroke	76	2.5	22	2.2	2.7	0.3	0.58	−0.2	0.82
Myocardial infarction	58	1.9	12	1.2	1.5	0.7	0.12	0.4	0.50
Heart failure	281	9.3	57	5.7	6.4	3.6	**0.0004**	2.9	**0.0092**
Diabetes mellitus	317	10.5	88	8.8	11.0	1.7	0.13	−0.5	0.73
Depression (ever before)	691	22.9	206	20.5	18.9	2.4	0.12	4.0	**0.0215**

Note

All results are based on 25 imputations of missing values. Numbers might not add up to total sample size due to rounding of multiple imputation results. Percentages might not add up to 100% due to rounding of percentages.

^a^ Adjusted to age distribution of cancer survivors cohort.

^b^ Diff. in proportions among controls minus survivors.

BC survivors with recurrence in contrast to disease‐free survivors were more often of a younger age (30‐49 years) at survey (11.8% vs 6.5%). They had less often stage I at diagnosis (34.2% vs 46.7%) and more often stage II (53.1% vs 45.7%) or stage III (12.7% vs 7.7%). Also, they reported less breast‐conserving therapy (55.6% vs 78.1%) and were more likely to be treated with chemotherapy (69.5% vs 58.6%). No significant differences were found with respect to axilla dissection, radio‐, or hormone therapy (Table 2).

**TABLE 2:** Description of breast cancer (BC) survivors (stage I to III at diagnosis) with and without recurrence after their breast cancer

	All BC survivors		BC survivors, recurrence		BC survivors, disease‐free		Difference^a^	
	n	%	n	%	n	%	%	*p* (CMH)
Total	3010	100	381	100	2629	100		
Mean age (SD)	65.3	(9.6)	64.6	(10.6)	65.4	(9.4)		**<.0001**
Age at survey								
30‐49	216	7.2	45	11.8	171	6.5	5.3	**.0002**
50‐59	597	19.8	67	17.6	530	20.2	−2.6	.24
60‐69	1083	36.0	128	33.6	955	36.3	−2.7	.30
70‐79	962	32.0	126	33.1	836	31.8	1.3	.62
80‐89	152	5.0	15	3.9	137	5.2	−1.3	.29
Tumor stage (UICC, TNM 6)								
I	1357	45.1	130	34.2	1227	46.7	−12.5	**<.0001**
II	1403	46.6	202	53.1	1201	45.7	7.4	**.0069**
III	250	8.3	49	12.7	201	7.7	5.0	**.0008**
Treatment								
Breast‐conserving^b^	2266	75.3	212	55.6	2054	78.1	−22.5	**<.0001**
Axilla dissection	2844	94.5	361	94.7	2483	94.4	0.3	.80
Radiotherapy	2528	84.0	320	83.9	2208	84	−0.1	.98
Chemotherapy	1805	60.0	265	69.5	1540	58.6	10.9	**<.0001**
Hormone therapy	1516	50.4	203	53.3	1313	49.9	3.4	.22

Note

All results are based on 25 imputations of missing values. Numbers might not add up to total number of BC survivors due to rounding of multiple imputation results. Percentages might not add up to 100% due to rounding of percentages.

^a^ Difference in proportions among BC survivors with recurrence minus disease‐free BC survivors.

^b^ Breast‐conserving therapy or mastectomy with reconstruction.

### 3.3 Prevalence of depression in BC survivors vs controls

Overall, mild/severe depression was found in 30.4% of BC survivors and in 23.8% of population controls (*p* = .0003), and severe depression only in 4.7% of BC survivors and 3.8% of controls (*p* = .22) (adjusted for age and education, data not shown).

**FIGURE 1:**
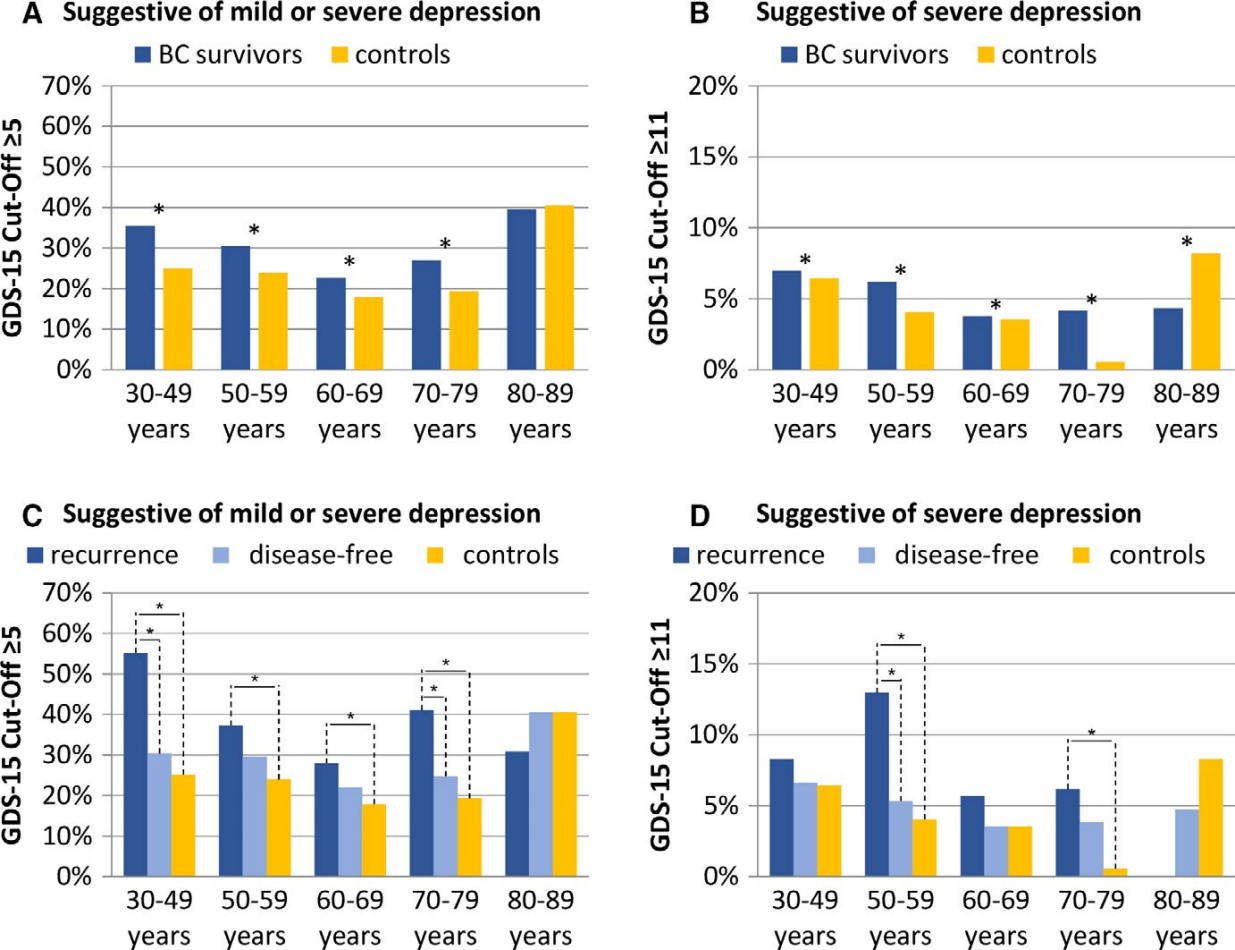
Prevalence of depression (according to GDS‐15) in BC survivors and controls; stratified by age (A, B) and by age and recurrence (C, D), adjusted for age and education. Asterisks (*) mark statistically significant differences in pairwise comparison (*p* < .05). The spans of the lines (C, D) indicate which subgroups differ significantly in pairwise comparison, for example, if the line spans three columns, it indicates a significant difference between BC survivors with recurrence and controls. Recurrence is defined as self‐report of recurrence, metastases, or another primary cancer after the diagnosis of the study cancer. All results are based on 25 imputations of missing values

When stratified by age, the prevalence of mild/severe depression was significantly higher in BC survivors than controls at all age groups <80 years, while at ≥80 years there was no difference between the groups (Figure 1A). The prevalence of severe depression was significantly higher in BC survivors than controls at all age groups <80 years. However, in age group 80‐89 years, the prevalence was significantly lower in BC survivors compared to controls (Figure 1B).

### 3.4 Prevalence of depression in BC survivors with recurrence vs disease‐free BC survivors

When further stratifying BC survivors according to recurrence status at survey, BC survivors with recurrence showed significantly higher prevalence of mild/severe depression than controls at all age groups ranging from 30 to 79 years. At age 30‐49 and 70‐79 years, BC survivors with a recurrence were also more likely to be depressed than disease‐free survivors. At age 80‐89, no significant differences were found between the groups (Figure 1C). For severe depression, a similar pattern was observed as for mild/severe depression. However, statistically significant differences were found only at age groups 50‐59 and 70‐79 years (Figure 1D).

### 3.5 Determinants of depression in BC survivors and controls

**FIGURE 2:**
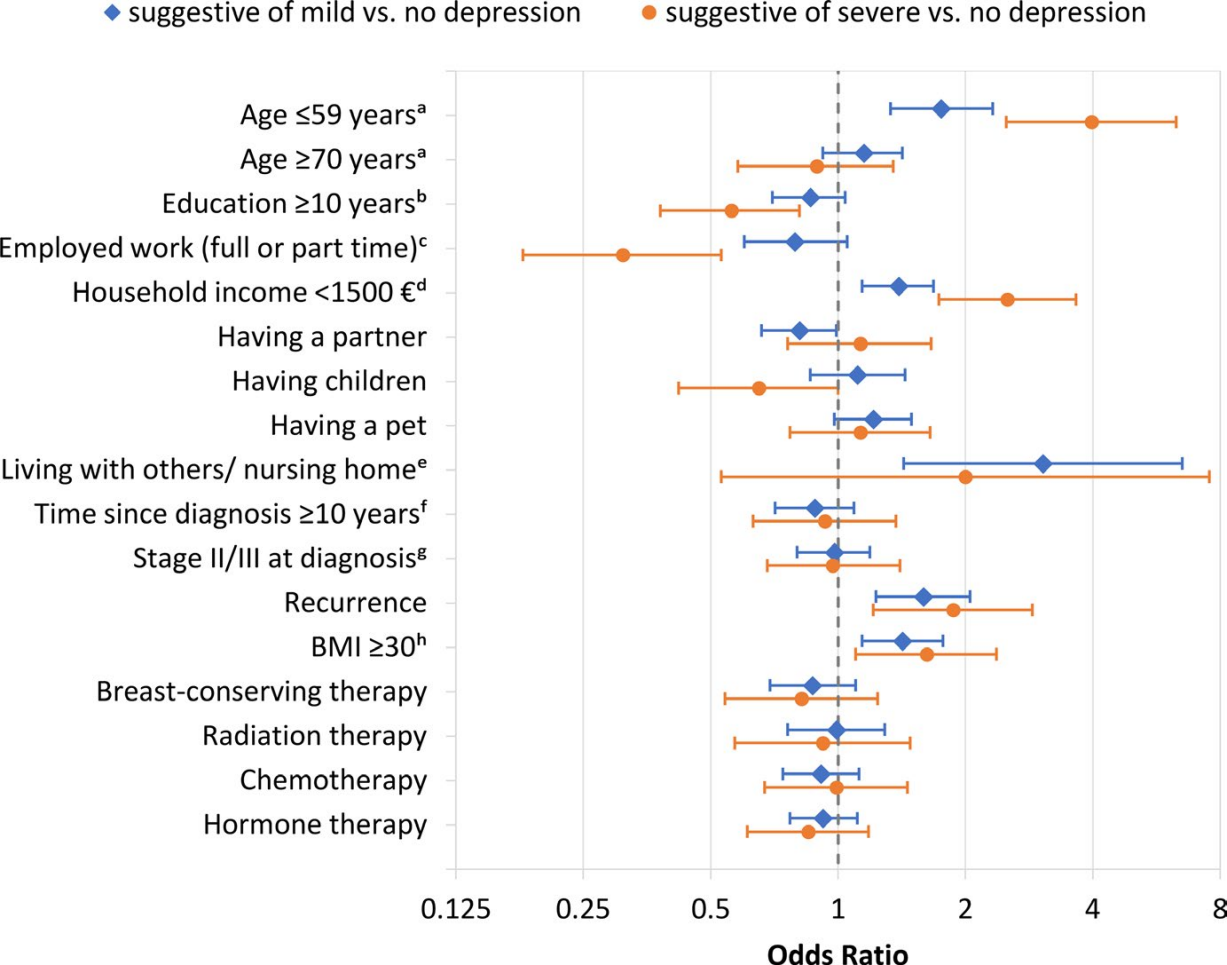
Predictors of depression in BC survivors (odds ratios with 95% confidence intervals). Reference groups: ^a^60‐69 y; ^b^<10 y; ^c^unemployed, housewife, or (early) retirement; ^d^≥1500€; ^e^living in an own household; ^f^5‐9 y; ^g^stage I; ^h^BMI < 30 kg/m^2^

In BC survivors, besides recurrence, age lower than 60 years, being obese (body mass index (BMI) of ≥30 kg/m^2^), and having a monthly household income of <€1500 were associated with both mild and severe depression (Figure 2). Additionally, the risk for a severe depression was also lower among higher educated (≥10 years) and employed (full or part‐time) BC survivors, while the risk for a mild depression was lower for BC survivors living in own household (vs living with others or in a nursing home). Time since diagnosis, stage (stage II or III vs stage I), treatments, having a permanent partner or having children were not significant risk factors.

When looking at factors associated with depression in controls, a similar pattern was found concerning age, employment, income, and BMI (Figure S2). However, in contrast to BC survivors, education was not statistically significantly associated with depression in this model. Instead, having a partner was associated with lower risk of mild and severe depression, and having children was associated with lower risk of severe depression. As a sensitivity analysis, we also applied the reduced model (without clinical variables) to the BC survivor sample. The results corresponded to those of the full model for BC survivors (data not shown).

## 4 DISCUSSION

With increasing numbers of long‐term BC survivors, it is essential to learn about persisting symptoms, and thus, potential supportive care needs of these individuals. This study demonstrated that even 5‐15 years after diagnosis, almost a third of BC survivors showed signs of a mild or a severe depression. Symptoms of a mild depression were more prevalent in BC survivors than in the general population of the same age. A recent population‐based Korean study found no difference between cancer survivors and controls.^23^ However, stage and recurrence were not controlled for in that study and subjects with chronic diseases were excluded from the start.

When stratified by age at survey, BC survivors reported slightly but significantly higher proportions of mild/severe and severe depression than controls in every age group <80 years. At 80‐89 years, BC survivors reported the same prevalence of mild/severe depression, and even lower prevalence of severe depression when compared to controls. Another German study on persons with various cancer entities also did not find any differences in the oldest studied age group (71‐85 years) compared to controls.^10^ High‐aged persons might perceive health problems as more “normal,” and thus, have a more optimistic view of their life situation.^10^ Besides, healthy survivor bias might play a role in this group, given the low number of BC survivors with recurrence in our study. As reported before, global health/ overall quality of life of disease‐free BC survivors aged ≥80 was better than in younger BC survivors and comparable to that of general population controls of same age.^5^

Many other previous studies on depression did not include long‐term survivors aged ≥80 years at survey. Given our results and the given high incidence rates of BC in women aged ≥70 years of ~183/100.000 globally,^1^ it seems important to expand the inclusion criteria in future studies and include participants of higher age.

In our study, risk of depression was higher in BC survivors who had a recurrence, metastasis or a secondary cancer after primary diagnosis. Recurrence in BC survivors is associated with distress^24^, ^25^ and hopelessness,^26^ and thus, can lead to depressive thoughts and feelings. However, as our study is based on cross‐sectional data, we cannot conclude on the direction of the discovered relationship. Depression is associated with lower adherence to screening or treatment,^27^ increased risk behavior like heavy smoking, or a lower immune response due to chronic stress, which could lead to a higher recurrence risk in depressive cancer survivors.^28^ Physiologically, cytokine changes induced by a recurrence can cause symptoms like fatigue, disturbed sleep, or a low energy level. These symptoms widely overlap with symptoms of depression.^28^ Another limitation of our study is that we were not able to further stratify the results by time since recurrence and/or by the number of recurrences. Case numbers of such subgroups would have been very small and the date of recurrence was often not reported.

Only personal and sociodemographic factors but not cancer‐ or treatment‐related variables other than recurrence were associated with depression in BC survivors. Previous results on the role of treatment factors for depression are mixed. A literature review showed that studies using bivariate analyses identified 10 cancer‐related risk factors for depression in BC survivors, while in multivariate analyses, only 3 factors remained significant.^13^ Thus, potential associations of treatment with depression might in fact be mediated by other variables considered in our multivariate analyses, like age or recurrence.

Having a partner was shown as a significant protecting factor for controls, but not for BC survivors. This is in contrast to previous multivariate studies, showing that partner support is associated with lower depression in BC survivors.^29^ However, we did not assess the quality of the relationship nor partner support in our study. The relation between marital status and poorer health‐related quality of life in long‐term BC survivors can be explained to a substantial extent by the social support given by the partner.^30^

Education was a significant factor for explaining severe depression in BC survivors. The protective role of education against the development of depression has been shown before.^31^ In healthy adults, lower education is also associated with a higher cancer fatalism and less information seeking.^32^ However, in our study, education was not associated with mild depression in BC survivors and neither mild nor severe depression in controls. For these groups, other socioeconomic factors might be more relevant, for example, household income, which was significant in both models. Our samples are generally elderly, born at a time when higher education for women was less common than today and marriage to a partner with better financial possibilities might have buffered the potential risk of a low education. Employed work was also associated with lower severe depression both in BC survivors and controls, which is in line with the literature.^33^

Obesity was associated with depression, both in BC survivors and controls. The relation between obesity and depression is supposed to be reciprocal, although the exact mechanism is not fully understood and both phenomena might also be based on common lifestyle factors like, for example, physical activity.^12^ Nevertheless, obesity can be a sign for clinicians to monitor their patients’ mood and refer to co‐treatment if necessary.^12^

Some limitations have to be considered when interpreting the results of this study. The GDS was constructed for a geriatric context and is not validated for younger participants. Therefore, the prevalence of depression in younger age groups should be interpreted in relation to the age‐stratified population norms and not on an absolute level. Also, the GDS assesses the affective and cognitive but not the somatic domains of depression.^21^ This reduces the potential overlap of depression with fatigue but might decrease the comparability with studies using other instruments. Further, the GDS is a self‐reported screening instrument that does not replace a diagnosis by a psychologist or psychiatrist. In our study, only 61.5% of BC survivors and controls with GDS scores suggestive of severe depression answered “yes” when directly asked whether they ever had a depression. Depression in BC survivors may be underdiagnosed and undertreated, either because the affected women do not disclose their emotional state or due to lack of recognition by clinicians.^6^, ^34^

Healthy survivor and/or participation bias could have led to an underrepresentation of BC survivors with poorer health in our study. As such, the prevalence of reported depression might be underestimated. Likewise, non‐participation among controls might also have introduced bias.^3^ Furthermore, we could not adjust for baseline depression of BC survivors and cannot draw any causal conclusions from our results based on the cross‐sectional data.

Strengths of the study include the population‐based recruitment of both samples with a comparable data collection mode. Both cohorts showed a variance in ages and sociodemographic variables, and the large sample size allowed for stratification according to age at survey and recurrence. There were no major differences between respondents and non‐respondents in the BC survivors’ cohort. We also conducted sensitivity analyses without excluding the small number of stage IV BC survivors and came to similar results.

A considerable number of long‐term BC survivors met the cut‐off for mild depression, although unlikely due to the primary cancer itself. Rather, recurrence explained most of the differences in depression prevalence between BC survivors and controls. Thus, when treating BC survivors for a recurrence, clinicians should be aware of the psychological effects and the existential meaning of being confronted with the illness again, refer to psychological care when needed, and normalize referral.

In conclusion, BC survivors, especially those who are younger and show additional risk factors for depression, should be monitored during follow‐up. Besides psychological support, information on financial aid and motivation to start/maintain some physical activity might be needed for this group.

## CONFLICT OF INTEREST

No conflict of interest.

## AUTHOR CONTRIBUTIONS

Daniela Doege: Formal analysis, methodology, visualization, writing ‐ original draft, review & editing. Melissa SY Thong: Methodology, writing – review & editing. Lena Koch‐Gallenkamp: Conceptualization, project administration, data curation, writing ‐ review & editing. Lina Jansen: Conceptualization, data curation, writing ‐ review & editing. Heike Bertram: Investigation, writing – review & editing. Andrea Eberle: Investigation, writing – review & editing. Bernd Holleczek: Investigation, writing – review & editing. Ron Pritzkuleit: Investigation, writing – review & editing. Annika Waldmann: Conceptualization, writing – review & editing. Sylke R. Zeissig: Investigation, writing – review & editing. Hermann Brenner: Conceptualization, funding acquisition, writing – review & editing. Volker Arndt: Conceptualization, funding acquisition, project administration, data curation, methodology, writing – review & editing, supervision.

## Supporting information

Supplementary Material

## Data Availability

Data available on request from the authors.

## References

[1] Bray F , Ferlay J , Soerjomataram I , Siegel RL , Torre LA , Jemal A . Global cancer statistics 2018: GLOBOCAN estimates of incidence and mortality worldwide for 36 cancers in 185 countries. CA Cancer J Clin. 2018;68:394‐424. 30207593 10.3322/caac.21492

[2] Parry C , Kent EE , Mariotto AB , Alfano CM , Rowland JH . Cancer survivors: a booming population. Cancer Epidemiol Biomark Prev. 2011;20:1996‐2005. 10.1158/1055-9965.EPI-11-0729 PMC3422885 21980007

[3] Arndt V , Koch‐Gallenkamp L , Jansen L , et al. Quality of life in long‐term and very long‐term cancer survivors versus population controls in Germany. Acta Oncol. 2017;56:190‐197. 28055266 10.1080/0284186X.2016.1266089

[4] Mols F , Vingerhoets AJJM , Coebergh JW , van de Poll‐Franse LV . Quality of life among long‐term breast cancer survivors: a systematic review. Eur J Cancer. 2005;41:2613‐2619. 16226458 10.1016/j.ejca.2005.05.017

[5] Doege D , Thong MS‐Y , Koch‐Gallenkamp L , et al. Health‐related quality of life in long‐term disease‐free breast cancer survivors versus female population controls in Germany. Breast Cancer Res Treat. 2019;175:499‐510. 30826935 10.1007/s10549-019-05188-x

[6] Reich M , Lesur A , Perdrizet‐Chevallier C . Depression, quality of life and breast cancer: a review of the literature. Breast Cancer Res Treat. 2008;110:9‐17. 17674188 10.1007/s10549-007-9706-5

[7] Maass SWMC , Roorda C , Berendsen AJ , Verhaak PFM , de Bock GH . The prevalence of long‐term symptoms of depression and anxiety after breast cancer treatment: a systematic review. Maturitas. 2015;82:100‐108. 25998574 10.1016/j.maturitas.2015.04.010

[8] Mitchell AJ , Ferguson DW , Gill J , Paul J , Symonds P . Depression and anxiety in long‐term cancer survivors compared with spouses and healthy controls: a systematic review and meta‐analysis. Lancet Oncol. 2013;14:721‐732. 23759376 10.1016/S1470-2045(13)70244-4

[9] Crespi CM , Ganz PA , Petersen L , Castillo A , Caan B . Refinement and psychometric evaluation of the impact of cancer scale. J Natl Cancer Inst. 2008;100:1530‐1541. 18957678 10.1093/jnci/djn340 PMC2586823

[10] Götze H , Friedrich M , Taubenheim S , Dietz A , Lordick F , Mehnert A . Depression and anxiety in long‐term survivors 5 and 10 years after cancer diagnosis. Support Care Cancer. 2020;28:211‐220. 31001695 10.1007/s00520-019-04805-1

[11] Luppa M , Sikorski C , Luck T , et al. Age‐ and gender‐specific prevalence of depression in latest‐life – systematic review and meta‐analysis. J Affect Disord. 2012;136:212‐221. 21194754 10.1016/j.jad.2010.11.033

[12] Luppino FS , de Wit LM , Bouvy PF , et al. Overweight, obesity, and depression: a systematic review and meta‐analysis of longitudinal studies. Arch Gen Psychiatry. 2010;67:220‐229. 20194822 10.1001/archgenpsychiatry.2010.2

[13] Bardwell WA , Fiorentino L . Risk factors for depression in breast cancer survivors: an update. Int J Clin Health Psychol. 2012;12:311‐331.

[14] Fang SY , Chang HT , Shu BC . The moderating effect of perceived partner empathy on body image and depression among breast cancer survivors. Psychooncology. 2015;24:1815‐1822. 26110591 10.1002/pon.3868

[15] Surbone A , Tralongo P . Categorization of cancer survivors: why we need it. J Clin Oncol. 2016;34:3372‐3374. 27458280 10.1200/JCO.2016.68.3870

[16] Northouse LL , Mood D , Kershaw T , et al. Quality of life of women with recurrent breast cancer and their family members. J Clin Oncol. 2002;20:4050‐4064. 12351603 10.1200/JCO.2002.02.054

[17] Arndt V , Merx H , Stegmaier C , Ziegler H , Brenner H . Persistence of restrictions in quality of life from the first to the third year after diagnosis in women with breast cancer. J Clin Oncol. 2005;23:4945‐4953. 16051947 10.1200/JCO.2005.03.475

[18] Thong MSY , Mols F , Doege D , van de Poll‐Franse L , Arndt V . Population‐based cancer survivorship research: experiences from Germany and the Netherlands. J Can Policy. 2018;15:87‐91.

[19] Yesavage JA , Sheikh JI . Geriatric Depression Scale (GDS): recent evidence and development of a shorter version. Clin Gerontol. 1986;5:165‐173.

[20] Gauggel S , Birkner B . Validität und Reliabilität einer deutschen Version der Geriatrischen Depressionsskala (GDS). Z Klin Psychol Psychother. 1999;28:18‐27.

[21] Smarr KL , Keefer AL . Measures of depression and depressive symptoms: Beck Depression Inventory‐II (BDI‐II), Center for Epidemiologic Studies Depression Scale (CES‐D), Geriatric Depression Scale (GDS), Hospital Anxiety and Depression Scale (HADS), and Patient Health Questionnaire‐9 (PHQ‐9). Arthritis Care Res. 2011;63:S454‐S466. 10.1002/acr.20556 22588766

[22] Clegg A , Barber S , Young J , Iliffe S , Forster A . The Home‐based Older People's Exercise (HOPE) trial: a pilot randomised controlled trial of a home‐based exercise intervention for older people with frailty. Age Ageing. 2014;43:687‐695. 24742587 10.1093/ageing/afu033 PMC4146519

[23] Lee SJ , Cartmell KB . Self‐reported depression in cancer survivors versus the general population: a population‐based propensity score‐matching analysis. Qual Life Res. 2020;29:483‐494. 31707694 10.1007/s11136-019-02339-x

[24] Syrowatka A , Motulsky A , Kurteva S , et al. Predictors of distress in female breast cancer survivors: a systematic review. Breast Cancer Res Treat. 2017;165:229‐245. 28553684 10.1007/s10549-017-4290-9 PMC5543195

[25] Andersen BL , Shapiro CL , Farrar WB , Crespin T , Wells‐Digregorio S . Psychological responses to cancer recurrence. Cancer. 2005;104:1540‐1547. 16118802 10.1002/cncr.21309 PMC2151214

[26] Brothers BM , Andersen BL . Hopelessness as a predictor of depressive symptoms for breast cancer patients coping with recurrence. Psychooncology. 2009;18:267‐275. 18702065 10.1002/pon.1394 PMC2743157

[27] DiMatteo MR , Lepper HS , Croghan TW . Depression is a risk factor for noncompliance with medical treatment: meta‐analysis of the effects of anxiety and depression on patient adherence. Arch Intern Med. 2000;160:2101‐2107. 10904452 10.1001/archinte.160.14.2101

[28] Spiegel D , Giese‐Davis J . Depression and cancer: mechanisms and disease progression. Biol Psychiatry. 2003;54:269‐282. 12893103 10.1016/s0006-3223(03)00566-3

[29] Talley A , Molix L , Schlegel RJ , Bettencourt A . The influence of breast cancer survivors' perceived partner social support and need satisfaction on depressive symptoms: a longitudinal analysis. Psychol Health. 2010;25:433‐449. 20397295 10.1080/08870440802582682

[30] Leung J , Smith MD , McLaughlin D . Inequalities in long term health‐related quality of life between partnered and not partnered breast cancer survivors through the mediation effect of social support. Psychooncology. 2016;25:1222‐1228. 27062092 10.1002/pon.4131

[31] Schlax J , Jünger C , Beutel ME , et al. Income and education predict elevated depressive symptoms in the general population: results from the Gutenberg health study. BMC Public Health. 2019;19:430. 31014301 10.1186/s12889-019-6730-4 PMC6480596

[32] Emanuel AS , Godinho CA , Steinman C , Updegraff JA . Education differences in cancer fatalism: the role of information‐seeking experiences. J. Health Psychol. 2018;23:1533‐1544. 27553609 10.1177/1359105316664129

[33] van der Noordt M , IJzelenberg H , Droomers M , Proper KI . Health effects of employment: a systematic review of prospective studies. Occup Environ Med. 2014;71:730‐736. 24556535 10.1136/oemed-2013-101891

[34] Hardman A , Maguire P , Crowther D . The recognition of psychiatric morbidity on a medical oncology ward. J Psychosom Res. 1989;33:235‐239. 2724199 10.1016/0022-3999(89)90051-2

