# Supplementary material for: Age‐specific prevalence and determinants of depression in long‐term breast cancer survivors compared to female population controls
Source: Cancer Med. 2020 Oct 6;9(22):8713–21. doi: 10.1002/cam4.3476 (PMC7666751; doi:10.1002/cam4.3476)
Supplement: Supplementary file 1 — Supplementary Material [file CAM4-9-8713-s001.docx]

**Age-specific prevalence and determinants of depression in long-term breast cancer survivors compared to female population controls**

Supplementary material


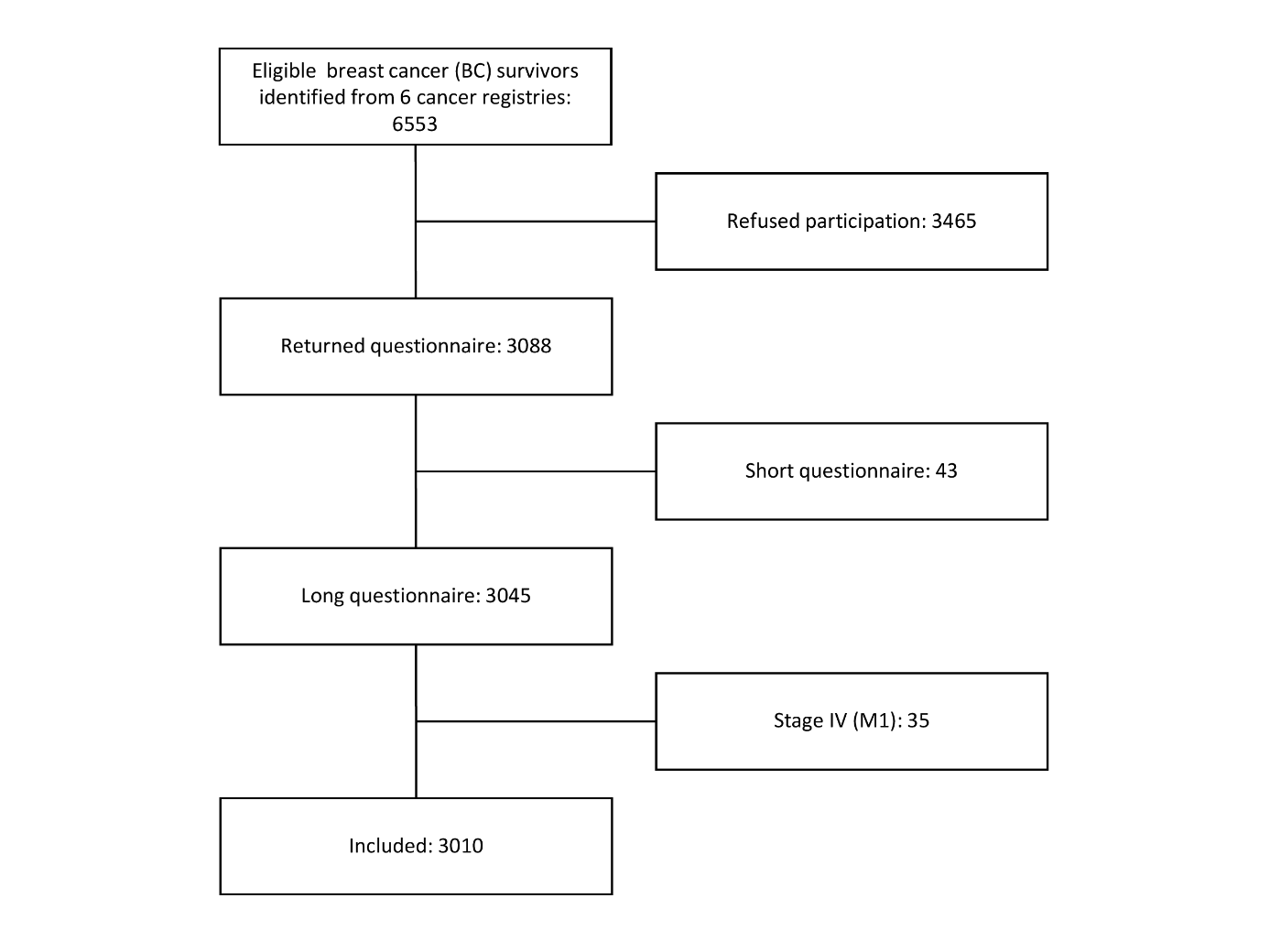
**Supplementary Figure 1: Flow Chart CAESAR+ (BC survivors)**


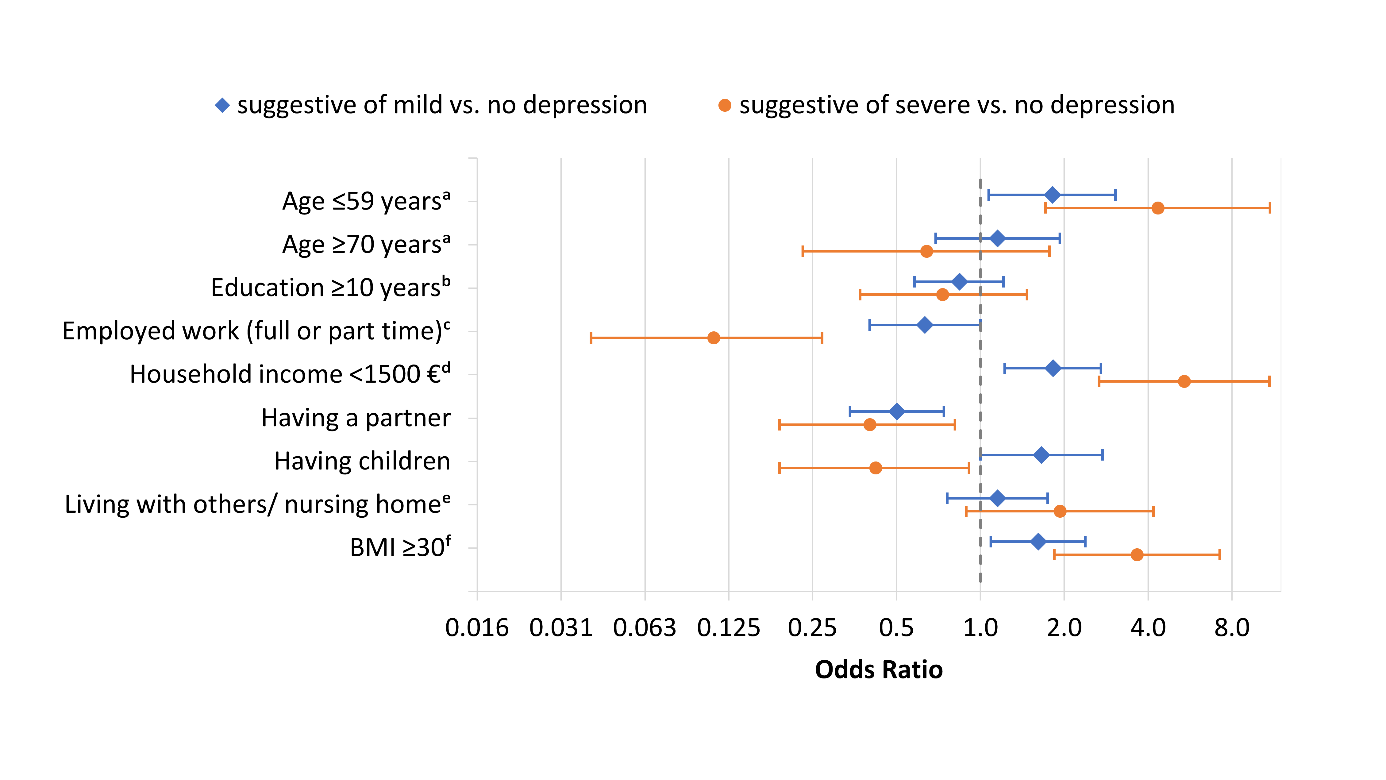


**Supplementary Figure 2: Predictors for depression in population controls (odds ratios with 95% confidence intervals)**

Reference groups: ^a^ 60-69 years; ^b^ <10 years; ^c^ unemployed, housewife or (early) retirement; ^d^ ≥1500 €; ^e^living in an own household; ^f^ BMI <30 kg/m².
